# Síndrome de Takotsubo: Uma Doença Recorrente?

**DOI:** 10.36660/abc.20200080

**Published:** 2020-04-06

**Authors:** Fábio Fernandes, Marcelo Westmoreland Montera

**Affiliations:** 1 Instituto do Coração Incor HC FMUSP São PauloSP Brasil Instituto do Coração - Incor HC FMUSP, São Paulo, SP - Brasil; 2 Hospital Pró-Cardíaco Rio de JaneiroRJ Brasil Hospital Pró-Cardíaco, Rio de Janeiro, RJ – Brasil

**Keywords:** Cardiomiopatia de Takotsubo/diagnóstico, Fatores Etários, Cardiomiopatia de Takotsubo/etiologia, Biomarcadores/sangue, Catecolaminas/sangue, Estrogênios/sangue

Vários termos (como Síndrome do Coração Feliz, Síndrome do Coração Partido e Cardiomiopatia de Takotsubo) têm sido utilizados para se referir à Síndrome de Takotsubo (STT), definida recentemente. O primeiro caso de STT foi descrito no Japão (Hiroshima City Hospital) em 1983 e um relato de cinco casos foi publicado em um livro de medicina japonês em 1990.^1^

Entretanto, em contraste com outras cardiomiopatias que geralmente não são transitórias por natureza, a STT é caracterizada por uma anormalidade temporária do movimento da parede do VE na ausência de feocromocitoma, miocardite e compartilha características comuns com sintomas semelhantes na apresentação da síndrome coronária aguda (SCA), anormalidades no ECG, biomarcadores cardíacos elevados, bem como uma mortalidade hospitalar comparável com infarto do miocárdio com supradesnivelamento do segmento ST (IAMCST) e sem supradesnivelamento do segmento ST (IAMSST), especificamente em termos de um forma microvascular da SCA.^2^ A Sociedade Europeia de Cardiologia (ESC) também estabeleceu o Critério Internacional de Diagnóstico Takotsubo ( *InterTAK Diagnostic Criteria* ), que implementa um algoritmo de diagnóstico e atribui uma pontuação à STT.^3^

Como os sintomas típicos de Takotsubo são o súbito aparecimento de dor torácica, falta de ar ou colapso, esses pacientes acreditam inicialmente que estão apresentando uma síndrome coronária aguda. Aproximadamente 1% a 3% de todos os pacientes que apresentam sintomas consistentes com SCA e são submetidos a angiografia coronária, são identificados como tendo STT.^4^

O alto nível de catecolamina parece ser devido à hiperativação do sistema hipotálamo-hipófise-adrenal em resposta a um gatilho exógeno, que nem sempre é facilmente reconhecido. Esses achados sugerem uma potencial interação coração-cérebro na fisiopatologia da STT, o papel do elo entre o coração e o cérebro e o fator desencadeante e o gênero, e as razões pelas quais essa síndrome apresenta fenótipos diferentes e, às vezes, apresenta recorrência.^5^

No início dos estudos, uma característica fundamental da síndrome de Takotsubo é a recuperação espontânea da fração de ejeção do VE, que retorna ao normal ou quase normal em quase todos os pacientes em um período variável de tempo (dias a semanas).^6^

Entretanto, a STT pode apresentar recorrência, com uma taxa de recidiva estimada em 1,8% por paciente ao ano.^7^ Como a recorrência não é frequente, isso impediu análises adicionais de preditores e desfechos. A recorrência da STT é definida como novas anormalidades do movimento da parede na ausência de doença coronariana obstrutiva após a recuperação dos eventos-índice da STT.^8^

O presente estudo publicado nos Arquivos Brasileiros de Cardiologia tem como objetivo analisar os principais fatores associados à recorrência da síndrome de Takotsubo. A taxa de recorrência global, considerando os estudos selecionados, foi de 3,8% em um tempo de seguimento que variou de 5 a 17 anos. Sexo feminino, tempo desde o primeiro episódio da síndrome, baixo IMC e obstrução média-ventricular foram relatados como possíveis preditores de recorrência da STT.^9^

Há um reconhecimento crescente das diferenças de gênero na apresentação, gatilhos, gravidade e complicações da STT. A literatura atual mostra uma predominância feminina no número de pacientes com STT (proporção de mulheres para homens 9:1).^7^ Várias explicações têm sido oferecidas, incluindo fatores como deficiência de estrogênio, gatilhos subjacentes e uma resposta do sistema nervoso autônomo aumentada.^6 , 7^

Em uma revisão sistemática do prognóstico em longo prazo e preditores de desfechos na Síndrome de Takotsubo, de 54 estudos que incluíram um total de 4.679 pacientes, durante um seguimento médio de 28 meses (intervalo interquartil: 23 a 34 meses), a taxa anual de mortalidade total foi de 3,5%, com uma taxa de recorrência anual de 1,0%. Uma análise de meta-regressão mostrou que a mortalidade total em longo prazo em cada estudo foi significativamente associada à idade mais avançada (p = 0,05), estressor físico (p = 0,0001) e à forma atípica de balonamento da STT (p = 0,009).

Os distúrbios neurológicos (taxa de risco: 1,76; p=0,048) e os transtornos psiquiátricos (taxa de risco: 1,77; p=0,033) emergiram como preditores independentes de recorrência. Esses achados sugerem que a STT precisa de seguimento rigoroso, devido à possibilidade de eventos adversos graves em longo prazo.^10 , 11^

A cardiomiopatia de Takotsubo tem 4 variantes anatômicas principais e uma categoria de outras variantes raras: variante apical, típica ou clássica, variante média-ventricular, variante basal, reversa ou invertida e a variante focal. A variante clássica e mais frequente da cardiomiopatia de Takotsubo geralmente afeta o ápice do ventrículo esquerdo. Entretanto, vários casos descreveram uma variante atípica.^12^ Acredita-se que as distribuições relativas dos adrenoceptores beta-2 determinem as diferentes variantes anatômicas. Um padrão variável da STT na recidiva é comum em até 20% dos casos de recorrência.^8^ Hipocinesia média-ventricular esquerda com hipercontratilidade basal e apical é relatada em 14,6% dos pacientes no Registro Internacional de Takotsubo.

Recentemente, o registro multicêntrico GEIST ( *German Italian Stress Cardiomyopathy* ) incluiu 749 pacientes consecutivos com STT, incluídos em 9 centros. No geral, a recorrência da STT foi documentada em 30 pacientes (4%), com mediana de seguimento de 830 dias. Fatores de risco cardiovasculares, como hipertensão arterial, foram significativamente maiores no grupo de recorrência. Curiosamente, em 14 pacientes (46%), a STT foi desencadeada por um novo estressor em comparação com o primeiro evento de STT (9 pacientes experimentaram um gatilho emocional e 5 pacientes experimentaram um gatilho físico) e até 2 recorrências de STT foram documentadas em 6% dos casos.^10^

Ainda existem muitas perguntas sem resposta sobre essa síndrome complexa. Curiosamente, nesta revisão, o uso de betabloqueadores ou outros medicamentos para insuficiência cardíaca não demonstrou reduzir a chance de recorrência. Kato et al.,^11^ observaram que 59,6% dos pacientes estavam em terapia regular com betabloqueadores na admissão relacionada à recorrência de STT, a maioria dos quais eram compostos seletivos beta 1 em 84,6%, sugerindo que antagonistas seletivos beta 1 podem não impedir a recorrência de STT e um tratamento ideal ainda precisa ser determinado.^11^
