# Supplementary material for: Síndrome de Takotsubo: Uma Doença Recorrente?
Source: Arq Bras Cardiol. 2020 Apr 6;114(3):484–5. [Article in Portuguese] doi: 10.36660/abc.20200080 (PMC7792729; doi:10.36660/abc.20200080)
Supplement: Translation

# Translation: Takotsubo Syndrome: A Recurrent Disease?

**Authors:** Fábio Fernandes, Marcelo Westmoreland Montera

**Affiliations:** 1 Brazil Instituto do Coração - Incor HC FMUSP, São Paulo, SP - Brazil; 2 Brazil Hospital Pró-Cardíaco, Rio de Janeiro, RJ – Brazil

**Keywords:** Takotsubo Cardiomyopathy/diagnosis, Takotsubo Cardiomyopathy/etiology, Biomarkers/blood, Catecholamines/blood, Estrogens/blood

Several terms (such as happy heart syndrome, broken heart syndrome, and takotsubo cardiomyopathy) have been used to refer to the recently defined Takotsubo Syndrome (TTS). The first case of TTS was described in Japan (Hiroshima City Hospital) in 1983 and a report of five cases was published in a Japanese medical textbook in 1990.^1^

However, in contrast to other cardiomyopathies that are usually not transient in nature, TTS is characterized by a temporary wall motion abnormality of the LV in the absence of pheochromocytoma, myocarditis and shares common features with acute coronary syndrome (ACS) similar symptoms at presentation, ECG abnormalities, elevated cardiac biomarkers, as well as a comparable in-hospital mortality with ST-segment elevation myocardial infarction (STEMI) and non-STEMI, specifically in terms of a microvascular ACS form.^2^The European Society of Cardiology (ESC) has also established the International Takotsubo Diagnostic Criteria (InterTAK Diagnostic Criteria), which implement a diagnostic algorithm and assign a score to TTS.^3^

As the typical Takotsubo symptoms are sudden onset of chest pain, breathlessness or collapse, these patients have an initial belief that they are experiencing acute coronary syndrome. Approximately 1%–3% of all patients who present with symptoms consistent with ACS and undergo coronary angiography, are identified to have TTS.^4^

The high level of catecholamine seems to be due to hyperactivation of the hypothalamus-pituitary gland-adrenal system in response to an exogenous trigger, which is not always easily recognized. These findings suggested a potential heart-brain interaction in the pathophysiology of TTS, the role of the link between the heart and brain and that of triggering factors and gender, and the reasons why this syndrome displays different phenotypes and sometimes recurs.^5^

At the beginning of the studies, a fundamental characteristic of Takotsubo is the spontaneous recovery of the LV ejection fraction, which returns to normal or near normal in all patients over a variable period of time (days to weeks).^6^

However, TTS may recur, with a recurrence rate estimated to be 1.8% per-patient year.^7^Because recurrence is not frequent, it precluded further analyses of predictors and outcomes. TTS recurrence is defined as new wall motion abnormalities in the absence of obstructive coronary disease after recovery of the index TTS events.^8^

In this study published in the Arquivos Brasileiros de Cardilogia aims to analyze the main factors associated with Takotsubo syndrome recurrence. The global recurrence rate, considering the selected studies, was 3.8% in a follow up that ranged from 5 to 17 years. Female gender, time from the first episode of the syndrome, low BMI and midventricular obstruction were reported as potential predictors of TTS recurrence.^9^

There is increasing recognition of gender differences in the presentation, triggers, severity, and complications of TTS. The current literature shows a female preponderance in the number of patients with TTS (female to male ratio 9:1).^7^Various explanations have been offered, including factors such as estrogen deficiency, underlying triggers, and a heightened autonomic nervous system response.^6 , 7^

In a systematic review and meta-regression of long-term prognosis and outcome predictors in Takotsubo Syndrome, of 54 studies that included a total of 4,679 patients, during a median follow-up of 28 months (interquartile range: 23 to 34 months), the annual rate of total mortality was 3.5% with an annual rate of recurrence of 1.0%. A meta-regression analysis showed that long-term total mortality in each study was significantly associated with older age (p = 0.05), physical stressor (p = 0.0001), and the atypical ballooning form of TTS (p = 0.009). Neurological disorders (hazard ratio: 1.76; p = 0.048) and psychiatric disorders (hazard ratio: 1.77; p = 0.033) emerged as independent predictors of recurrence. These findings suggest that TTS needs a strict follow-up, due to the possibility of severe adverse events over the long term.^10 , 11^

Takotsubo cardiomyopathy has 4 main anatomic variants and a category of other rare variants: Apical, typical, or classic variant, Midventricular variant, Basal, reverse, or inverted variant and the Focal variant. The classic and most frequent variant of Takotsubo cardiomyopathy usually affects the left ventricular apex. However, several cases have described an atypical variant.^12^Relative distributions of the beta-2 adrenoceptors are believed to determine the different anatomic variants. A variable TTS pattern at recurrence is common in up to 20% of recurrence cases.^8^Mid-left ventricular hypokinesia with basal and apical hypercontractility is reported for 14.6% of patients in the International Takotsubo Registry.

Recently, the multicenter GEIST (German Italian Stress Cardiomyopathy) Registry included 749 consecutive patients with TTS, enrolled from 9 centers. Overall, TTS recurrence was documented in 30 patients (4%) at a median follow-up of 830 days. Cardiovascular risk factors, such as arterial hypertension were significantly higher in the recurrence group. Interestingly, in 14 patients (46%), TTS was triggered by a new stressor compared with the first TTS event (9 patients experienced an emotional trigger, and 5 patients experienced a physical trigger) and up to 2 TTS recurrences were documented in 6% of cases.^10^

There remain many unanswered questions regarding this complex syndrome. Interestingly, in this review the use of betablockers or other heart failure medications was not proved to reduce the chance of recurrence. Kato et al.,^11^showed that 59.6% of patients were on regular betablocker therapy on admission related to TTS recurrence, most of which were beta 1-selective compounds in 84.6%, suggesting that beta 1-selective antagonists might not prevent TTS recurrence and an optimal treatment still needs to be determined.^11^
